# Association of genetic predisposition to immune-related biomarkers with premenstrual disorders and symptom severity

**DOI:** 10.1186/s12888-026-08443-w

**Published:** 2026-08-05

**Authors:** Yijie Luo, Jiantong Sun, Min Chen, Qing Pan, Ting Pan, Jin Huang, Huan Song, Yuchen Li, Donghao Lu

**Affiliations:** 1 Mental Health Center, West China Hospital, China Health Center for Mental Disorders, Sichuan University, 37 Guoxue Ln, Chengdu, Chengdu, Sichuan 610041 China; 2 West China School of Medicine, West China Hospital, Sichuan University, Chengdu, 610041 China; 3 Department of Thoracic Surgery, Institute of Thoracic Oncology, West China Hospital, Sichuan University, Chengdu, China; 4 Health Management Center, Innovation Institute for Integration of Medicine and Engineering, West China Hospital, General Practice Medical Center, Sichuan University, Chengdu, China; 5 Department of Anesthesiology and West China Biomedical Big Data Center, West China Hospital, Sichuan University, Chengdu, China; 6 Institute of Environmental Medicine, Karolinska Institutet, Stockholm, Sweden

**Keywords:** Inflammation, Polygenic risk score, Premenstrual disorder, Premenstrual syndrome, White blood cell

## Abstract

**Background:**

Premenstrual disorders (PMD), including premenstrual syndrome (PMS) and the more severe premenstrual dysphoric disorder (PMDD), are burdensome conditions with unclear mechanisms. While inflammation is implicated, biomarker research is challenged by inconsistent findings and cyclical fluctuations. This study utilized polygenic risk scores (PRS) to investigate the association between lifelong genetic predisposition to altered immune-related cell counts and PMD risk and symptom severity.

**Methods:**

This cross-sectional study included 1,730 Chinese college students from the Care of Premenstrual Emotion cohort. PRS were calculated for seven immune biomarkers: white blood cell (WBC), basophil, eosinophil, lymphocyte, monocyte, neutrophil, and C-reactive protein. Probable PMD cases and symptom severity (total, physical, affective) were assessed using a modified Calendar of Premenstrual Experiences. Generalized estimating equations estimated risk ratios (RR) and β-coefficients, adjusting for confounders.

**Results:**

Overall, 424 (24.5%) participants met probable PMD criteria, including 379 (21.9%) with PMS and 45 (2.6%) with PMDD. The PRS for WBC showed a suggestive, non-significant association with diagnosis (RR, 1.08; 95% CI, 1.00–1.18; *P* = 0.062). No other immune PRS showed significant associations. A higher PRS for WBC was associated with physical symptom severity (β = 0.05; 95% CI, 0.00–0.10; *P* = 0.047). This association was stronger in women with symptom onset at menarche (β = 0.47; 95% CI, 0.15–0.78; *P* = 0.008). The highest quintile of PRS for WBC exhibited greater severity across all domains versus the lowest, suggesting a possible graded pattern. Item-level analyses suggested signals for limb swelling, increased appetite, and food cravings.

**Conclusions:**

The PRS for WBC may be associated with premenstrual symptom severity, specifically physical domains. These findings suggest that inflammatory liability may contribute to the physical symptom burden of PMD, but replication in prospectively phenotyped cohorts with detailed gynecologic and medical comorbidity data is needed.

**Trial registration:**

Not applicable.

**Supplementary Information:**

The online version contains supplementary material available at https://doi.org/10.1186/s12888-026-08443-w.

## Graphical Abstract

**Figure:**
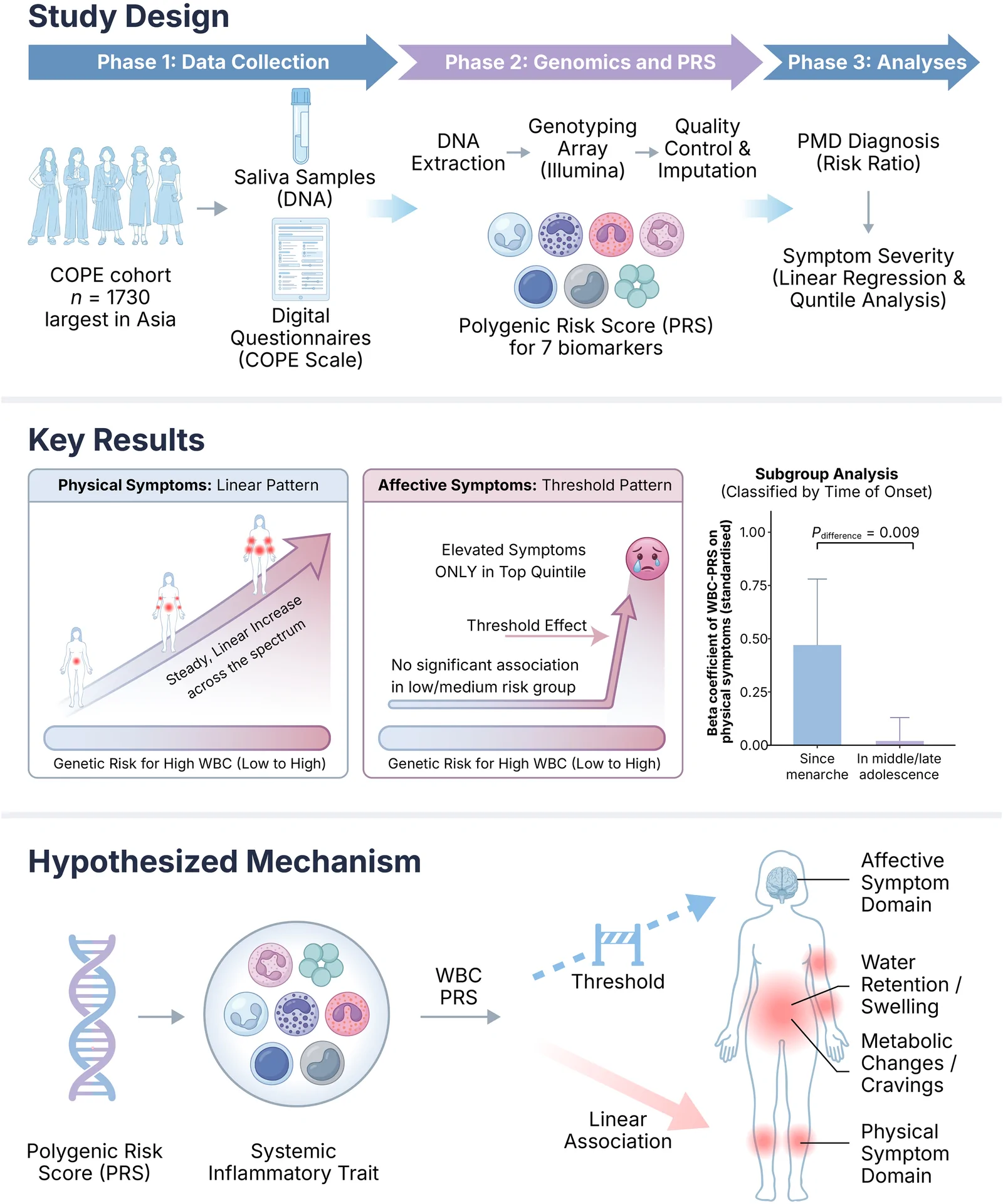

## Introduction

Premenstrual disorders (PMD), including premenstrual syndrome (PMS) and the more severe premenstrual dysphoric disorder (PMDD), are characterized by a recurrent cluster of affective and physical symptoms, which emerge during the late luteal phase and resolve shortly after the onset of menstruation [1]. PMD are highly prevalent, affecting 24.4% of women globally [2]. Furthermore, PMD are linked to negative health outcomes, including suicidal behavior [3], perinatal depression [4], and cardiovascular diseases [5]. Research has established that the core mechanism is an abnormal neurobiological response to normal hormonal fluctuations, rather than a hormonal imbalance itself [6, 7]. However, what triggers this aberrant sensitivity remains unknown, with potential factors like inflammation [8] and genetic polymorphisms [9].

A growing body of evidence implicates the immune system and inflammatory responses in the pathogenesis of PMD. Our epidemiological findings support this link, demonstrating that childhood asthma [10], an allergic-inflammatory condition, and childhood obesity [11], which could be considered as a chronic inflammatory disease [12, 13], are both associated with an increased risk of developing PMD in young adulthood. Moreover, few studies have linked premenstrual symptoms to pro-inflammatory markers such as tumor necrosis factor-alpha (TNF-α), interleukins (ILs), and C-reactive protein (CRP) [14, 15], which play a role in the neuroinflammatory processes [8]. White blood cell count is a routinely measured leukocyte-based indicator of inflammatory activity and may reflect chronic low-grade inflammation [16].

However, these findings are inconsistent, potentially due to challenges including relatively small sample sizes. For example, a study among 897 adolescent girls found a positive association between high-sensitivity C-reactive protein (hs-CRP) and the presence of PMS [17], while a longitudinal study of 41 women concluded that PMS was not accompanied by significant changes in hs-CRP levels [18]. Findings on the anti-inflammatory cytokine IL-10 are also contradictory: one study of 277 women found that its levels were significantly higher in those with PMS [15], whereas another study in 52 female soccer players observed a negative correlation between IL-10 and depressive symptoms, a core feature of PMD [19]. Moreover, these conflicting results may stem from the inherent limitations of measuring peripheral biomarkers at a single timepoint, which reflects transient, state-dependent immune activity across the menstrual cycle [20]. The polygenic risk score (PRS) for immune-related biomarkers, however, can serve as a proxy for a woman’s lifelong genetic predisposition to an altered immune-related profile, which is independent of cyclical or acute changes. This genetic approach provides a novel opportunity to investigate whether an innate, stable alteration in immune function contributes to the risk and manifestation of PMD.

Therefore, using data from a large cohort of young women with comprehensive questionnaire and genotyping data, our study aimed to investigate the association between the genetic predisposition for seven key immune-related biomarkers and the risk of PMD as well as their distinct symptom dimensions.

## Methods

### Study participants

This study was conducted on a sub-population of the Care of Premenstrual Emotion (COPE) cohort, a prospective study of female college students recruited via cluster dormitory sampling at Sichuan University initiated in 2021. At baseline, the study was designed to concurrently collect comprehensive questionnaire information (demographics, health behaviors, social experiences) and biospecimens for the study of PMD.

Eligible participants for the present genetic analysis were female college students enrolled in the Care of Premenstrual Emotion (COPE) cohort who provided electronic informed consent, completed the baseline PMD and covariate questionnaires, and provided a saliva sample for genotyping. As of September 2024, a total of 2008 participants had been enrolled. After excluding 15 participants with missing questionnaire fields or invalid responses, 1918 (96.2%) completed saliva sampling. After excluding participants with sequencing failures (*n* = 7), hormonal medication usage (*n* = 64), or genotype quality-control failure (*n* = 117), 1730 participants were included in the final analysis. A flowchart of the sample selection process is shown in Supplementary Fig. 1. All participants provided electronic informed consent, and the study received ethical clearance from the Institutional Review Board of West China Hospital, Sichuan University (No. 2023 − 179).

### Genotyping, quality control and calculation of PRS

DNA extracted from saliva samples was genotyped using the Infinium Chinese Genotyping Array BeadChip (Illumina WeGene V3) and subjected to stringent quality control (QC) to exclude discordant samples and low-quality variants (call rate < 98%, HWE *P* < 1 × 10⁻⁶), followed by imputation using the 1000 Genomes Project Phase 3 East Asian reference panel. Post-imputation, variants with INFO < 0.9 or MAF < 0.01 were removed, and LD-pruned genotypes were used to calculate principal components (PCs) to control for population stratification. For PRS calculation, we utilized summary statistics for seven immune-related biomarkers (basophil, eosinophil, lymphocyte, monocyte, neutrophil, and WBC counts, as well as CRP) from large-scale East Asian GWAS [21, 22]; these data were harmonized with the target dataset after removing low-quality and ambiguous variants. The PRS was derived using the “clumping and thresholding” (C + T) method [23] in PLINK (v1.90b6.21) following standard protocols [24], and a robust composite PRS was constructed by performing PCA on scores derived from seven different P-value thresholds, with the first principal component standardized for subsequent analyses [25]. For each biomarker, the PRS was oriented so that higher values represented greater genetic predisposition to higher biomarker levels or counts. Further details are provided in Supplementary Methods.

### Assessment of premenstrual symptoms and PMD

PMD were assessed with a modified version of the Calendar of Premenstrual Experiences (COPE) scale. The scale required participants to retrospectively rate 8 affective symptoms (4 for mood instability, 4 for depressed mood) and 19 physical symptoms (4 for pain/discomfort, 6 for gastrointestinal/appetite changes, 6 for general somatic issues, and 3 for cognitive/sleep disturbances) on a four-point severity scale [26] (detailed in Supplementary Tables 2 and 3), followed by the impact of PMD symptoms on daily activities and interpersonal relationships. The scale has been validated in other populations using prospective daily symptom diaries, with a positive predictive value of 80% [27, 28]. We also computed a total symptom score (range: 27–108), an affective symptom score, and a physical symptom score, all of which were converted to Z-scores for analysis.

As described elsewhere [29], a probable PMD diagnosis was defined based on four criteria: (a) presence of both affective and physical symptoms; (b) moderate-to-severe overall severity or functional impairment; (c) specific timing of symptom onset and resolution relative to menses; and (d) symptom absence post-menstruation. To shed light on subtypes, PMD were further grouped as PMS or probable PMDD. Women were categorized as having probable PMDD if they met the following criteria [30]: (a) at least one of four severe emotional symptoms; (b) at least five symptoms from a defined list of 11 groups; and (c) moderate-to-severe functional impairment. To distinguish between cases that began to have symptoms since menarche and those that started in middle or late adolescence, PMD were also categorized by the self-reported timing of symptom onset. This subgrouping reflected symptom-onset timing rather than participants’ current age.

### Covariates

To control for population stratification (ancestry-related allele-frequency differences that can create spurious genotype-phenotype associations), the first four genetic PCs were included as covariates in all regression models together with the PRS exposure. The number of PCs was selected based on a scree plot analysis (Supplementary Fig. 2). Data on demographics were collected through baseline questionnaires, such as age [31], school, and education level [32, 33]. Health and lifestyle variables included body mass index (BMI), which was calculated from self-reported weight and height [34]; age at menarche [35], referring to the first occurrence of menstruation in females [36]; and alcohol consumption, defined as any use in the past month [37, 38]. Finally, adverse childhood experiences (ACEs) were assessed using the Chinese version of the World Health Organization Adverse Childhood Experiences-International Questionnaire [39], with exposure defined by meeting predefined frequency thresholds [40, 41]. Depressive and anxiety symptoms were assessed using the Patient Health Questionnaire-9 (PHQ-9) and the General Anxiety Disorder-7 (GAD-7), respectively.

### Statistical analysis

Baseline characteristics of the study population were summarized to compare women with and without PMD. Continuous variables were assessed for normality using the Shapiro-Wilk test. As the key variables did not follow a normal distribution (*P* < 0.05), they were presented as medians and interquartile ranges. Differences between groups were subsequently evaluated using the non-parametric Wilcoxon rank-sum test. Categorical variables were presented as counts and percentages (n, %), and the difference was assessed using Pearson’s chi-squared test.

To examine the association between the PRS for immune-related biomarkers and PMD, we conducted generalized estimating equation (geeglm) models with a Poisson distribution and log link to estimate risk ratios (RRs). We established four models: Model 1 adjusted for the first four PCs; Model 2 (primary model) adjusted for the first four PCs and age. To illustrate potential influence from demographic characteristics and known risk factors for PMD, two additional models were developed: Model 3 additionally adjusted for school and education; Model 4 further adjusted for BMI, ACEs, alcohol consumption, and age at menarche. To examine the association with symptom severity scores, we used geeglm models with a Gaussian distribution (equivalent to a linear model). In such analysis, both the PRS and the symptom scores were standardized to z-scores, meaning the resulting beta (β) coefficient represents the unit change of standard deviation (SD) in symptom severity per one SD increase in the PRS. To examine the pattern of association without assuming linearity, we repeated the analyses with the PRS categorized into quintiles, setting the lowest quintile as the reference. The linear trend test modeled PRS quintile as an ordinal variable (1–5) and was used as an exploratory dose-response assessment rather than evidence of a strict threshold.

Additional analyses were performed on immune-related biomarkers with statistical and clinical significance, including subgroup and interaction tests. To examine heterogeneity, we performed subgroup analyses by PMD subtype (PMS vs. PMDD; different timings of symptom onset), and stratified analyses for comorbidity of depression or anxiety. To compare the magnitude of association across subgroups, we used two-sample Z-tests comparing coefficients and standard errors from the stratified models; beta coefficients were used for symptom-severity analyses, and log-risk-ratio coefficients were used for PMD-risk analyses. To test for effect modification by depression or anxiety, we introduced a multiplicative interaction term (e.g., PRS×Depression) into the regression models. As a final exploratory step, we investigated the association of the PRS for WBC with each symptom item. Because seven immune-related PRS were selected a priori rather than screened from a broad biomarker database, nominal two-sided P-values are reported; FDR-adjusted P-values were calculated for reference across the seven immune-related biomarkers. All statistical analyses were conducted in R (v4.3.3), with a two-sided P-value < 0.05 considered statistically significant.

## Results

### Characteristics of study participants

The final analysis included 1730 participants, comprising 424 (24.5%) women who met the criteria for a probable PMD. Participants had a median age of 20 years (interquartile range, 19–22), a median age at menarche of 12 years, and a median BMI of approximately 20 kg/m². As shown in Table 1, women with PMD reported a significantly higher prevalence of ACEs (58% vs. 51%; *P* = 0.02) and anxiety symptoms (15% vs. 6.7%; *P* < 0.01) compared to those without PMD. Additionally, women with PMD had a marginally earlier age at menarche (*P* = 0.046).

**Table 1:** Comparisons of baseline characteristics between women with and without PMD

Characteristic	Without PMD(*n* = 1306)^a^	With PMD(*n* = 424)^a^	*P* ^b^
Age (years)	20 (19, 22)	20 (19, 22)	0.61
Age at Menarche (years)	12 (12, 13)	12 (11, 13)	0.05
BMI (kg/m^2^)^c^	20.07 (18.75, 21.83)	20.08 (18.73, 21.78)	> 0.99
BMI Category (kg/m^2^)^c^			0.33
≥13 to < 18	155 (15%)	44 (14%)	
≥18 to < 24	777 (75%)	256 (79%)	
≥24	104 (10%)	25 (7.7%)	
ACE^d^			0.02
Any ACE	526 (51%)	190 (58%)	
No ACE	510 (49%)	135 (42%)	
Alcohol Consumption			0.08
Ever	696 (67%)	235 (72%)	
Never	340 (33%)	90 (28%)	
School			0.09
Medicine	558 (87%)	191 (83%)	
Others	82 (13%)	40 (17%)	
Education			0.24
Graduate	270 (21%)	99 (23%)	
Undergraduate	1,036 (79%)	325 (77%)	
Irregular Menstrual Cycle^e^			0.74
Irregular	249 (19%)	84 (20%)	
Regular	1,057 (81%)	340 (80%)	
Depression^f^			0.24
Yes	37 (3.7%)	16 (5.3%)	
No	950 (96%)	286 (95%)	
Anxiety^g^			< 0.001
Yes	88 (6.7%)	65 (15%)	
No	1,218 (93%)	359 (85%)	

^a^ Median (Q1, Q3); n (%)

^b^ Pearson’s Chi-squared test for categorical variables; Wilcoxon rank sum test for continuous variables

^c^ BMI: Body Mass Index

^d^ ACE: Adverse Childhood Experiences

^e^ Regular cycles are defined as those with ≤ 7-day variation

^f^ A cut-off score of 10 was used for the Patient Health Questionnaire-9 (PHQ-9)

^g^ A cut-off score of 10 was used for the General Anxiety Disorder-7 (GAD-7)

### Association between PRS for immune-related biomarkers and PMD

The correlations among the seven immune-related biomarkers are shown in Supplementary Fig. 3. Mean PRS values were small relative to their SDs, indicating substantial genetic-score overlap between women with and without PMD. A positive yet statistically non-significant trend was suggested between PRS for WBC and PMD (RR = 1.08; 95% CI, 1.00–1.18; *P* = 0.062; Table 2). The PRS for the other six immune-related biomarkers showed no significant association with PMD (RRs ranging from 0.94 to 1.07 in Model 2). Similar results were yielded with additional adjustments; for WBC, the RR was 1.08 (95% CI, 0.95–1.23; *P* = 0.253) in Model 3 and 1.07 (95% CI, 0.93–1.23; *P* = 0.353) in Model 4 (Supplementary Table 4). Comparable yet non-significant patterns were observed in the analysis by PRS quintiles (Supplementary Fig. 4), with all P for trend > 0.05.

**Table 2:** Association between genetic susceptibility of immune-related biomarkers and PMD risk

Trait	Without PMD (*n* = 1306)	With PMD (*n* = 424)	Model 1^b^		Model 2^c^	
	PRS (mean ± sd)^a^	PRS (mean ± sd)^a^	RR (95% CI)^d^	*P*	RR (95% CI)^d^	*P*
C-reactive protein	-0.02 ± 2.31	-0.01 ± 2.26	0.99 (0.91–1.08)	0.869	0.99 (0.91–1.08)	0.838
Basophil count	-0.09 ± 2.32	0.03 ± 2.29	1.03 (0.95–1.13)	0.435	1.03 (0.95–1.13)	0.433
Eosinophil counts	-0.07 ± 2.26	-0.15 ± 2.33	0.94 (0.86–1.03)	0.212	0.94 (0.86–1.03)	0.206
Lymphocyte count	0.02 ± 2.36	0.03 ± 2.22	1.01 (0.94–1.10)	0.749	1.01 (0.94–1.10)	0.744
Monocyte count	0.00 ± 2.30	0.08 ± 2.40	1.02 (0.94–1.11)	0.664	1.02 (0.94–1.11)	0.655
Neutrophil count	-0.04 ± 2.40	0.19 ± 2.20	1.07 (0.99–1.16)	0.081	1.07 (0.99–1.16)	0.082
White blood cell count	-0.04 ± 2.41	0.24 ± 2.39	1.08 (1.00-1.18)	0.062	1.08 (1.00-1.18)	0.062

^a^ PRS: Polygenic risk scores

^b^ Model 1 adjusted for four population principal components

^c^ Model 2 adjusted for four population principal components and age

^d^ RR: Risk ratio; CI: Confidence interval

### Association between PRS for immune-related biomarkers and PMD symptom severity

A higher PRS for WBC was associated with greater physical symptom severity (β = 0.05; 95% CI, 0.00–0.10; *P* = 0.047; Table 3), although this association was not significant for total symptom severity (β = 0.05; 95% CI, -0.00–0.10; *P* = 0.061) or affective symptom severity (*P* = 0.146). No associations were found for the PRS for the other six immune-related biomarkers.

**Table 3:** Association between genetic susceptibility of immune-related biomarkers and symptom severity

Trait	Overall Symptoms^a^		Affective Symptoms^a^		Physical Symptoms^a^	
	Beta (95% CI)^b^	*P*	Beta (95% CI)^b^	*P*	Beta (95% CI)^b^	*P*
C-reactive protein	0.01 (-0.04, 0.05)	0.807	0.03 (-0.02, 0.08)	0.249	-0.01 (-0.06, 0.04)	0.655
Basophil count	-0.01 (-0.06, 0.05)	0.831	-0.00 (-0.06, 0.05)	0.865	-0.01 (-0.06, 0.04)	0.821
Eosinophil counts	-0.03 (-0.09, 0.02)	0.205	-0.02 (-0.08, 0.03)	0.390	-0.04 (-0.09, 0.01)	0.157
Lymphocyte count	-0.01 (-0.06, 0.04)	0.762	-0.01 (-0.06, 0.04)	0.641	-0.00 (-0.05, 0.04)	0.878
Monocyte count	0.02 (-0.03, 0.07)	0.434	0.01 (-0.04, 0.05)	0.779	0.03 (-0.02, 0.07)	0.293
Neutrophil count	0.04 (-0.01, 0.08)	0.131	0.03 (-0.02, 0.07)	0.247	0.04 (-0.01, 0.08)	0.109
White blood cell count	0.05 (-0.00, 0.10)	0.061	0.04 (-0.01, 0.09)	0.146	0.05 (0.00, 0.10)	0.047

^a^ All models were adjusted for age and the first four population principal components

^b^ Beta: Beta coefficient; CI: Confidence interval. Beta coefficient from linear regression indicates the change in standardized symptom score per one standard deviation increase in the polygenic risk score (PRS)

**Fig. 1:**
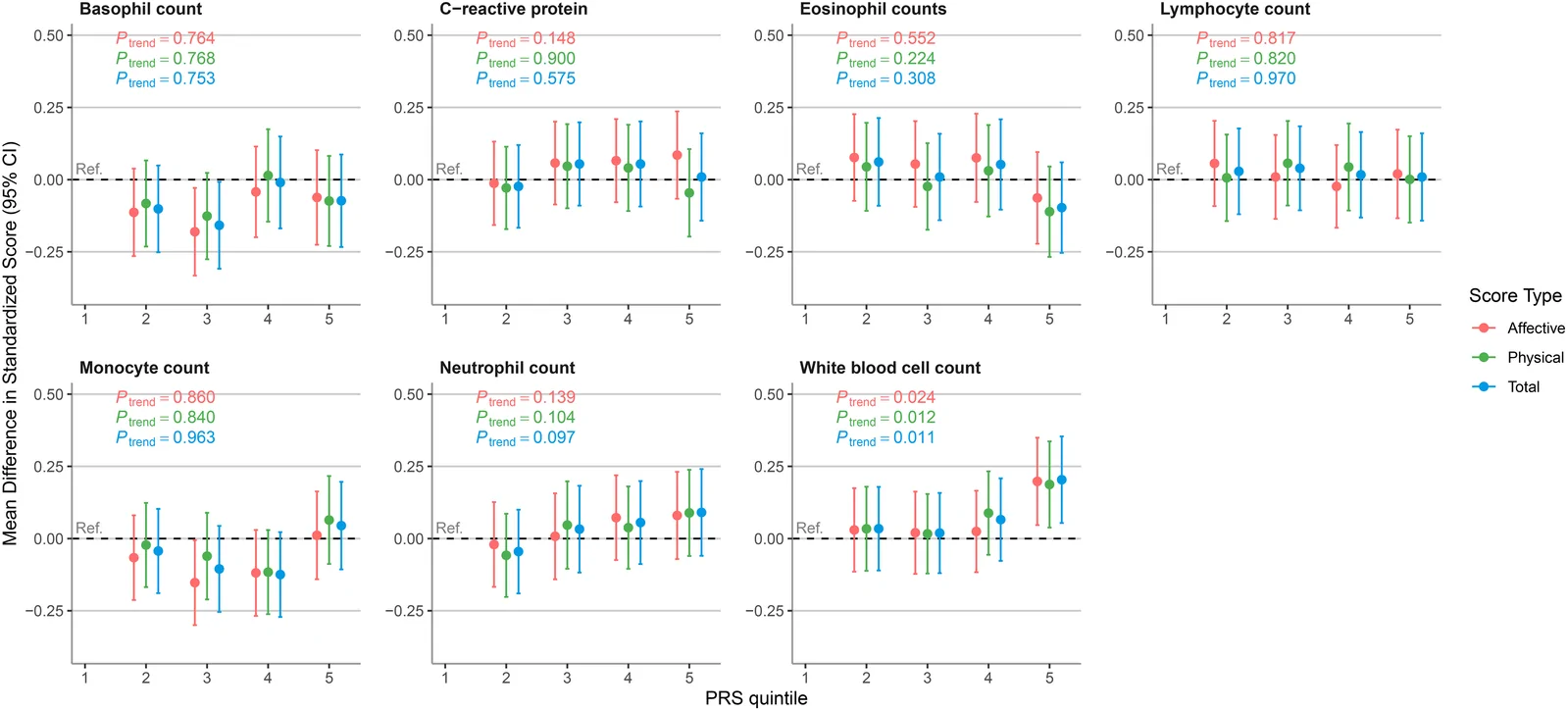
Association between polygenic risk score for immune-related biomarkers in quintiles and PMD symptom severity. PRS: polygenic risk scores. Points show the beta coefficients from regression models comparing quintiles 2–5 to the lowest quintile (reference). This coefficient was adjusted for four population principal components and age. Symptom scores were converted to Z-scores

When the PRS for WBC was stratified into quintiles, the test for linear trend was statistically significant (*P* for trend = 0.02, 0.01, 0.01 for affective, physical, total scores, respectively; Fig. 1): the highest quintile showed significantly greater symptom severities compared to the lowest (total: β = 0.20; 95% CI, 0.05–0.35; *P* = 0.008; affective: β = 0.20; 95% CI, 0.05–0.35; *P* = 0.010; physical:β = 0.19; 95% CI, 0.04–0.34; *P* = 0.014).

In exploratory item-level analyses of the 19 physical symptoms, the PRS for WBC was positively associated with premenstrual limb swelling (*P* = 0.03), with weaker signals for increased appetite (*P* = 0.057) and food cravings (*P* = 0.074) (Supplementary Table 2). No associations were observed between the PRS for WBC and any of the affective symptoms, although a borderline association was indicated for premenstrual depression and low mood (*P* = 0.060) (Supplementary Table 3).

### Additional analyses

Based on these findings, additional analyses were performed to assess the relationship between the PRS for WBC and physical symptoms in different subgroups. In this case-only analysis, the association between the PRS for WBC and physical symptoms was stronger in PMD women whose symptoms began at menarche (β = 0.47; 95% CI, 0.15–0.78; *P* = 0.008), but not in those with a later onset (β = 0.02; 95% CI, -0.09–0.13; *P* = 0.737) (Table 4). In contrast, the association was not significant when stratified by PMD severity (PMS vs. PMDD).

**Table 4:** Association between genetic susceptibility of white blood cell and physical symptom severity in PMD subtypes^a^

	Women, *N*	PRS^b^	Beta (95% CI)^c^	*P*
PMD classified by severity				
PMS	379	0.24 ± 2.38	0.05 (-0.05, 0.16)	0.302
PMDD	45	0.24 ± 2.48	-0.15 (-0.46, 0.17)	0.360
*P* for difference				0.229
PMD classified by time of onset				
Since menarche	33	0.05 ± 2.18	0.47 (0.15, 0.78)	0.008
In middle/late adolescence	391	0.25 ± 2.41	0.02 (-0.09, 0.13)	0.737
*P* for difference				0.009

^a^ PMD: Premenstrual disorders

^b^ PRS: Polygenic risk scores (mean ± standard deviation)

^c^ Beta: Beta coefficient; CI: Confidence interval. Betas represent the change in standardized physical symptom score per one standard deviation increase in PRS, adjusted for four population principal components and age. *P* for difference was calculated using a two-sample Z-test comparing coefficients and standard errors from the stratified models

We found a comparable association of the PRS for WBC with PMD between participants with and without comorbid anxiety (P-for-interaction > 0.05; Supplementary Table 6). Similarly, the association with physical symptom severity was not clearly modified by depression or anxiety (P-for-interaction > 0.05; Supplementary Table 7).

## Discussion

In this study of 1730 young female adults in China, we systematically investigated the association between the genetic predisposition for seven immune-related biomarkers and PMD. While no significant associations were found between the PRS for any of the seven immune-related biomarkers and risk of PMD, the PRS for WBC showed a suggestive association. A more consistent pattern was observed for symptom dimensions: the PRS for WBC was associated with physical symptom severity, whereas the association with affective symptom severity was not statistically significant. Given the small effect estimates and the overlap in PRS distributions between women with and without PMD, these findings should be interpreted as preliminary group-level evidence rather than as an individual-level risk classification signal.

To our knowledge, this is the first study to link the PRS for WBC to the physical symptoms of PMD. This provides genetic evidence consistent with phenotypic studies linking peripheral inflammation to physical symptoms in mood disorders. Our finding aligns with longitudinal research identifying elevated WBC-related biomarkers as predictors of major depression [42]. While they focused on the predictive value of inflammatory trajectories, our results suggest that the PRS for WBC may similarly be associated with premenstrual physical symptomatology. This is further supported by longitudinal research finding that women with higher baseline total white blood cell counts experienced a more rapid increase in “physical complaints” of depression over time [43].

Compared with the pattern observed for the PRS for WBC, the null findings for the other six immune-related PRS require careful interpretation. These null findings stand in contrast to existing studies based on direct phenotypic measurements. To date, several studies examining direct immune phenotypes have reported positive associations. A large cross-sectional study of midlife women found that elevated hs-CRP levels were significantly associated with premenstrual mood symptoms, abdominal cramps/back pain, and appetite cravings/weight gain [44]. Similarly, another study reported that girls with both PMS and primary dysmenorrhea had significantly higher serum hs-CRP and a higher neutrophil-to-lymphocyte ratio [17]. This discrepancy likely highlights the distinction between a stable, lifelong genetic predisposition (measured by PRS) and a fluctuating, state-dependent immune phenotype (measured by serum markers). Although our approach cannot capture acute inflammatory changes, it allows us to examine whether stable genetic liability to immune-related traits is associated with PMD-related symptom burden.

The quintile analyses suggested a graded association between the PRS for WBC and symptom severity, with the pattern appearing more consistent for physical symptoms than for affective symptoms. Our analysis of individual symptoms showed the PRS for WBC was specifically associated with premenstrual limb swelling and marginally associated with increased appetite and food cravings. These findings are consistent with the biological roles of inflammatory processes in fluid retention [45, 46] and metabolic changes [47], although these pathways could not be directly tested in the present study. In contrast, the pathways linking peripheral inflammation to central affective symptoms are more complex, often involving slower-acting central nervous system cascades and neurotransmitter changes [48]. This may indicate that immune-related genetic liability is more closely reflected in physical symptom burden, whereas links with affective symptoms may be weaker, less direct, or more dependent on additional neurobiological pathways.

Notably, the association between the PRS for WBC and physical symptom severity appeared most pronounced among women whose symptoms began at menarche. This finding should be interpreted cautiously because the subgroup was small (*n* = 33). Nevertheless, it is broadly consistent with epidemiological evidence linking pubertal timing, particularly age at menarche, to premenstrual symptom burden and PMD/PMS risk [49]. These findings suggest that the perimenarchal period may be a relevant window for PMD vulnerability, but prospective cohorts initiated before or around menarche are needed to clarify whether immune-related genetic liability contributes to early symptom trajectories. Furthermore, while research directly linking WBC count to physical symptoms of PMD is lacking, studies on other immune-related biomarkers also support our findings. For example, serum levels of IL-4 and IL-12 have been positively associated with physical symptom scores of PMD [15]. From a translational perspective, since PRS is fixed at birth and needed to be tested only once, it may help generate hypotheses about early vulnerability to physical symptom trajectories. Identifying adolescent girls with high PRS for WBC could allow for anticipatory guidance and management of physical symptoms before they become chronic and burdensome PMD.

It remains unclear whether PRS for WBC possesses sufficient utility as a standalone screening tool. Future prospective cohorts initiated pre-puberty should validate its predictive value for physical symptom trajectories. Although SSRIs and hormonal approaches remain clinically important [50–53], our findings are not sufficient to support treatment selection by the PRS for WBC. Future studies should use careful phenotyping to distinguish PMD from dysmenorrhea and other gynecologic or medical causes of perimenstrual physical symptoms.

A major strength of this study is the use of a deeply phenotyped cohort with available genetic data and the application of validated tools for a multi-dimensional assessment of PMD symptoms. However, several limitations must be acknowledged. First, GWAS summary statistics for immune-related biomarkers in East Asian populations are very limited, which may affect the predictive power of the PRS used in our study. While this is a known challenge, our PRS was constructed from the largest available East Asian GWAS to maximize validity. Second, although the COPE-based assessment enabled structured evaluation of probable PMD and dimensional symptom burden in a large genetically characterized cohort, it does not replace a prospectively confirmed DSM-based diagnosis. Diagnostic literature emphasizes that PMS/PMDD diagnosis ideally requires prospective daily symptom ratings across two menstrual cycles, careful medical and psychiatric history, physical examination, and assessment for premenstrual worsening of another medical condition [54, 55]. This may have introduced phenotype misclassification, including possible inclusion of women whose cyclical affective distress or disability was secondary to primary gynecologic or medical conditions. In particular, primary dysmenorrhea is common in young women, is mechanistically linked to increased prostaglandin production, and is effectively treated with nonsteroidal anti-inflammatory drugs [56]. Earlier NSAID literature also cautioned that evidence for NSAIDs was much stronger for dysmenorrhea than for PMS [57]. Third, detailed physician-diagnosed gynecologic and medical comorbidities, such as dysmenorrhea, endometriosis, autoimmune disease, and acute infection, were not available; we therefore could only exclude hormonal medication usage and assess psychiatric comorbidity using PHQ-9 and GAD-7. Fourth, inflammatory indices are pleiotropic and may reflect etiologic liability, downstream consequences of symptoms and disability, or shared comorbid processes [58, 59]. Therefore, our findings should be viewed as preliminary genetic evidence for an immune-related contribution to PMD symptom severity, not proof of an inflammatory cause of PMD or PMDD. Fifth, our study population consists of Chinese female university students, which may limit the generalizability of our findings to other populations. However, the prevalence of PMD observed in our cohort is largely consistent with reports from other population-based studies [60], which supports the generalizability of our findings within this demographic.

Taken together, this study provides preliminary evidence that the PRS for WBC is associated with the severity of premenstrual physical symptoms. Our results suggest that WBC-related inflammatory liability may be associated with the physical burden of PMD, but replication in prospective cohorts with more stringent PMD/PMDD phenotyping and richer comorbidity data is needed before clinical or causal conclusions can be drawn.

## Supplementary Information

Below is the link to the electronic supplementary material.

Supplementary Material 1

## Data Availability

The datasets generated and/or analysed during the current study are available from the corresponding author on reasonable request.

## Abbreviations

PMD: Premenstrual disorders
PMS: Premenstrual syndrome
PMDD: Premenstrual dysphoric disorder
PRS: Polygenic risk score
WBC: White blood cell
CRP: C-reactive protein
hs-CRP: High-sensitivity C-reactive protein
ACEs: Adverse childhood experiences
GWAS: Genome-wide association study
QC: Quality control
PCs: Principal components
RR: Risk ratio
CI: Confidence interval
SD: Standard deviation
