# Supplementary material for: Association of genetic predisposition to immune-related biomarkers with premenstrual disorders and symptom severity
Source: BMC Psychiatry. 2026 Aug 5;26:860. doi: 10.1186/s12888-026-08443-w (PMC13729386; doi:10.1186/s12888-026-08443-w)
Supplement: Supplementary file 1 [file 12888_2026_8443_MOESM1_ESM.docx]

**Supplementary Material**

*Association of genetic predisposition to immune-related biomarkers with premenstrual disorders and symptom severity*

Contents

[Supplementary Methods: Details for Polygenic Risk Score (PRS) calculation	2](#_Toc217581289)

[Genotyping, Imputation, and Quality Control	2](#_Toc217581290)

[Calculation of PRS	2](#_Toc217581291)

[References	2](#_Toc217581292)

[Supplementary Figure 1. Flowchart of this study.	3](#_Toc217581293)

[Supplementary Figure 2. Scree plot of immune-related biomarkers.	4](#_Toc217581294)

[Supplementary Figure 3. A heatmap of the correlation matrix for polygenic risk scores of various inflammation markers.	5](#_Toc217581295)

[Supplementary Figure 4. Association between polygenic risk score of immune-related biomarkers in quintiles and PMD.	6](#_Toc217581296)

[Supplementary Table 1. Summary of genome-wide association studies (GWAS) used for polygenic risk score (PRS) calculation.	7](#_Toc217581297)

[Supplementary Table 2. Association between WBC PRS and Individual Physical Symptom Severity.^a^	8](#_Toc217581298)

[Supplementary Table 3. Association between WBC PRS and Individual Affective Symptom Severity.a	9](#_Toc217581299)

[Supplementary Table 4. Association between genetic susceptibility of immune-related biomarkers and PMD risk with additional adjustments.	9](#_Toc217581300)

[Supplementary Table 5. Association between genetic susceptibility of immune-related biomarkers and PMD subtypes.^a^	10](#_Toc217581301)

[Supplementary Table 6. Association between genetic susceptibility of white blood cell and PMD, stratified by depression and anxiety.^a^	11](#_Toc217581302)

[Supplementary Table 7. Association between genetic susceptibility of white blood cell and physical symptom severity in PMD, stratified by depression and anxiety.^a^	12](#_Toc217581303)

## Supplementary Methods: Details for Polygenic Risk Score (PRS) calculation

### Genotyping, Imputation, and Quality Control

DNA was extracted from participants’ saliva samples and genotyped using the Infinium Chinese Genotyping Array BeadChip Illumina WeGene V3 Arrays, with an initial count of 727, 349 SNPs. A stringent quality control (QC) process was applied. We excluded samples exhibiting sex-genotype discordance. Variants with abnormal heterozygosity, a call rate below 98%, or significant deviation from Hardy-Weinberg Equilibrium (*P* < 1 × 10⁻⁶) were removed. To increase genomic coverage, we performed genotype imputation using the East Asian reference panel from the 1000 Genomes Project Phase 3, with 10,159,805 SNPs after imputation. We further removed variants with an imputation information score (INFO) < 0.9 or a minor allele frequency (MAF) < 0.01. Then we performed linkage disequilibrium (LD) pruning on the quality-controlled genotypes (r² < 0.25, 200 kb window, 50-variant step). Using the LD-pruned variants, we evaluated cryptic relatedness and excluded closely related individuals ($\hat{\pi}$ > 0.125). The remaining unrelated samples were used for before calculating principal components (PCs) as covariates for controlling population stratification. Number of SNPs that passed all QC filters are detailed in Supplementary Table 1.

### Calculation of PRS

To obtain genome-wide association study (GWAS) summary statistics for immune-related biomarkers, we selected the largest available studies conducted exclusively in East Asian populations ^1,2^. Following a systematic screening, seven immune-related biomarkers were chosen for PRS calculation: basophil count, eosinophil count, lymphocyte count, monocyte count, neutrophil count, white blood cell (WBC) count, and CRP, a widely-used and accessible indicator for inflammation (Supplementary Table 1). All summary statistics were standardized to the GRCh37 genome build. The base GWAS data then underwent QC; variants with low imputation quality (INFO < 0.8), low MAF (< 0.01), or strand ambiguity (A/T and G/C palindromic SNPs) were removed. The quality-controlled base and target datasets were then harmonized by aligning alleles and removing mismatched variants.

PRS was calculated using the “clumping and thresholding” (C+T) method in PLINK (v1.90b6.21) ^3^following standard protocols ^4^. SNPs were clumped using an r² threshold of 0.1 within a 250-kb window. The PRS for each woman $i$ was calculated using the formula $\text{PRS}_{i}=\sum_{j=1}^{N} \beta_{j}G_{ij}$, where $N$ is the number of SNPs included in the score, $\beta_{j}$ is the effect size of the risk allele for SNP $j$ from the source GWAS, and $G_{ij}$ is the dosage of the risk allele (0, 1, or 2) for SNP $j$ carried by woman $i$. To build a robust genetic instrument, we calculated the PRS at seven different P-value thresholds (P = 0.001, 0.05, 0.1, 0.2, 0.3, 0.4, 0.5) and then performed a principal component analysis (PCA) on these seven scores and used the first principal component (PC1) as the final composite PRS^5^. This final PRS was standardized to a Z-score (mean=0, standard deviation=1) for use in all subsequent association analyses.

### References

1.	Sakaue S, Kanai M, Tanigawa Y, et al. A cross-population atlas of genetic associations for 220 human phenotypes. *Nat Genet*. 2021;53(10):1415-1424. doi:10.1038/s41588-021-00931-x

2.	Chen CY, Chen TT, Feng YCA, et al. Analysis across Taiwan Biobank, Biobank Japan, and UK Biobank identifies hundreds of novel loci for 36 quantitative traits. *Cell Genom*. 2023;3(12):100436. doi:10.1016/j.xgen.2023.100436

3.	Chang CC, Chow CC, Tellier LC, Vattikuti S, Purcell SM, Lee JJ. Second-generation PLINK: rising to the challenge of larger and richer datasets. *Gigascience*. 2015;4:7. doi:10.1186/s13742-015-0047-8

4.	Choi SW, Mak TSH, O’Reilly PF. Tutorial: a guide to performing polygenic risk score analyses. *Nat Protoc*. 2020;15(9):2759-2772. doi:10.1038/s41596-020-0353-1

5.	Coombes BJ, Ploner A, Bergen SE, Biernacka JM. A principal component approach to improve association testing with polygenic risk scores. *Genet Epidemiol*. 2020;44(7):676-686. doi:10.1002/gepi.22339

## Supplementary Figure 1. Flowchart of this study.

## Supplementary Figure 2. Scree plot of immune-related biomarkers.

The scree plots show the proportion of variance explained by each principal component derived from the polygenic risk scores constructed at different p-value thresholds.

## Supplementary Figure 3. A heatmap of the correlation matrix for polygenic risk scores of various inflammation markers.

PRS: polygenic risk scores.

Values represent Pearson correlation coefficients between the PRS of different immune-related biomarkers.

**Supplementary Figure 4. Association between polygenic risk score of immune-related biomarkers in quintiles and PMD.**

PRS: polygenic risk scores; RR: risk ratio; CI: confidence interval.

This coefficient was adjusted for four population principal components and age. Symptom scores were converted to Z-scores.

## Supplementary Table 1. Summary of genome-wide association studies (GWAS) used for polygenic risk score (PRS) calculation.

| GCST ID | Trait | Sample Ancestry | First Author | Publication Date | PubMed ID | Alleles Used for Scoring^a^ |
| --- | --- | --- | --- | --- | --- | --- |
| GCST90018730 | C-reactive protein | 83025 East Asian | Sakaue S | 30 September 2021 | 34594039 | 380, 7969, 14049, 22878, 29963, 35628, 40398 |
| GCST90018726 | Basophil count | 91908 East Asian | Sakaue S | 30 September 2021 | 34594039 | 564, 8423, 14383, 22860, 29703, 35488, 40334 |
| GCST90018733 | Eosinophil counts | 93063 East Asian | Sakaue S | 30 September 2021 | 34594039 | 694, 8658, 14624, 23121, 30064, 35785, 40437 |
| GCST90018742 | Lymphocyte count | 95717 East Asian | Sakaue S | 30 September 2021 | 34594039 | 635, 8841, 14684, 23175, 30049, 35708, 40441 |
| GCST90018747 | Monocyte count | 95119 East Asian | Sakaue S | 30 September 2021 | 34594039 | 744, 9087, 15091, 23854, 30693, 36318, 40906 |
| GCST90018748 | Neutrophil count | 82810 East Asian | Sakaue S | 30 September 2021 | 34594039 | 653, 8803, 14959, 23490, 30159, 35850, 40584 |
| GCST90278673 | White blood cell count | 246970 East Asian | Chen CY | 16 November 2023 | 38116116 | 1588, 12363, 18546, 27417, 33965, 39148, 43372 |

^a^ Numbers of SNPs used to construct PRS at a series of p-value thresholds (0.001, 0.05, 0.1, 0.2, 0.3, 0.4, 0.5), from which the robust principal component PRS was derived.

## Supplementary Table 2. Association between WBC PRS and Individual Physical Symptom Severity.^a^

| Symptom Item | Beta (95% CI)^b^ | *P* |
| --- | --- | --- |
| Bloating | 0.01 (-0.04, 0.06) | 0.741 |
| Breast tenderness | 0.04 (-0.01, 0.09) | 0.105 |
| Dizziness | 0.02 (-0.03, 0.07) | 0.524 |
| Headache | 0.01 (-0.04, 0.06) | 0.755 |
| Hot flashes | 0.03 (-0.02, 0.09) | 0.226 |
| Nausea | 0.03 (-0.02, 0.08) | 0.246 |
| Limb swelling | 0.05 (0.01, 0.10) | 0.030 |
| Pimples | 0.01 (-0.04, 0.06) | 0.597 |
| Diarrhea | 0.03 (-0.02, 0.08) | 0.181 |
| Food cravings | 0.04 (-0.00, 0.09) | 0.074 |
| Palpitations | 0.04 (-0.01, 0.10) | 0.139 |
| Increased appetite | 0.05 (-0.00, 0.10) | 0.057 |
| Decreased appetite | 0.04 (-0.01, 0.09) | 0.159 |
| Fatigue | 0.04 (-0.01, 0.09) | 0.128 |
| Insomnia | 0.02 (-0.03, 0.07) | 0.435 |
| Forgetfulness | 0.02 (-0.04, 0.07) | 0.562 |
| Abdominal cramps | 0.04 (-0.01, 0.09) | 0.125 |
| Lower back pain | 0.01 (-0.03, 0.06) | 0.555 |
| Difficulty concentrating | 0.04 (-0.02, 0.09) | 0.180 |

^a^ All models were adjusted for age and the first four population principal components.

^b^ Beta: Beta coefficient; CI: Confidence interval. Beta represents the change in the standardized individual symptom score per one standard deviation increase in the standardized PRS for White blood cell count.

## Supplementary Table 3. Association between WBC PRS and Individual Affective Symptom Severity.a

| Symptom Item | Beta (95% CI)^b^ | *P* |
| --- | --- | --- |
| Anxiety and stress | 0.01 (-0.04, 0.06) | 0.669 |
| Irritability | 0.04 (-0.01, 0.09) | 0.142 |
| Emotional sensitivity | 0.04 (-0.01, 0.09) | 0.101 |
| Mood swings | 0.02 (-0.03, 0.07) | 0.481 |
| Tearfulness | 0.01 (-0.04, 0.06) | 0.699 |
| Anger outbursts | 0.04 (-0.01, 0.10) | 0.088 |
| Depression and low mood | 0.05 (-0.00, 0.10) | 0.060 |
| Loss of interesting daily activities | 0.04 (-0.01, 0.09) | 0.144 |

^a^ All models were adjusted for age and the first four population principal components.

^b^ Beta: Beta coefficient; CI: Confidence interval. Beta represents the change in the standardized individual symptom score per one standard deviation increase in the standardized PRS for White blood cell count.

## Supplementary Table 4. Association between genetic susceptibility of immune-related biomarkers and PMD risk with additional adjustments.

| Trait | PRS (mean ± sd)^a^ | | Model 3^b^ | | Model 4^c^ | | Reference |
| --- | --- | --- | --- | --- | --- | --- | --- |
|  | Without PMD (n = 1306)^d^ | With PMD (n = 424)^d^ | RR (95% CI)^e^ | *P* | RR (95% CI)^e^ | *P* |  |
| C-reactive protein | -0.02 ± 2.31 | -0.01 ± 2.26 | 0.99 (0.90-1.08) | 0.808 | 1.00 (0.90-1.10) | 0.973 | Sakaue et. al, 2021 |
| Basophil count | -0.09 ± 2.32 | 0.03 ± 2.29 | 1.03 (0.93-1.14) | 0.541 | 1.04 (0.93-1.17) | 0.494 | Sakaue et. al, 2021 |
| Eosinophil counts | -0.07 ± 2.26 | -0.15 ± 2.33 | 0.94 (0.86-1.04) | 0.242 | 0.94 (0.84-1.05) | 0.296 | Sakaue et. al, 2021 |
| Lymphocyte count | 0.02 ± 2.36 | 0.03 ± 2.22 | 1.04 (0.93-1.15) | 0.514 | 1.03 (0.93-1.15) | 0.543 | Sakaue et. al, 2021 |
| Monocyte count | 0.00 ± 2.30 | 0.08 ± 2.40 | 0.99 (0.83-1.17) | 0.887 | 0.98 (0.83-1.15) | 0.829 | Sakaue et. al, 2021 |
| Neutrophil count | -0.04 ± 2.40 | 0.19 ± 2.20 | 1.06 (0.96-1.16) | 0.237 | 1.08 (0.97-1.20) | 0.184 | Sakaue et. al, 2021 |
| White blood cell count | -0.04 ± 2.41 | 0.24 ± 2.39 | 1.08 (0.95-1.23) | 0.253 | 1.07 (0.93-1.23) | 0.353 | Chen et. al, 2023 |

^a^ PRS: polygenic risk scores

^b^ Model 3 was adjusted for four population principal components, age, school and education.

^c^ Model 4 was additionally adjusted for body mass index, adverse childhood experiences, alcohol consumption, and age at menarche.

^d^ PMD: Premenstrual disorders

^e^ RR: risk ratio; CI: confidence interval

For covariates with missing values in Supplementary Table 2, multiple imputation was conducted using chained equations (MICE), where imputation was carried out with 5 imputations and 5 iterations using the predictive mean matching (PMM) method, and results were pooled across imputed datasets.

## Supplementary Table 5. Association between genetic susceptibility of white blood cell count and PMD subtypes.^a^

|  | Women, N | PRS^b^ | RR (95% CI)^c^ | *P* |
| --- | --- | --- | --- | --- |
| PMD classified by severity | | | | |
| PMS | 379 | 0.24 ± 2.38 | 1.08 (0.99, 1.18) | 0.081 |
| PMDD | 45 | 0.24 ± 2.48 | 1.14 (0.85, 1.53) | 0.368 |
| *P* for difference |  |  |  | 0.727 |
| PMD classified by time of onset | | | | |
| Since menarche | 33 | 0.05 ± 2.18 | 1.03 (0.76, 1.38) | 0.870 |
| In middle/late adolescence | 391 | 0.25 ± 2.41 | 1.09 (1.00, 1.19) | 0.055 |
| *P* for difference |  |  |  | 0.693 |

^a^ PMD: Premenstrual disorders

^b^ PRS: Polygenic risk scores (mean ± standard deviation)

^c^ RR: Risk ratio; CI: Confidence interval. RRs were adjusted for four population principal components and age.

## Supplementary Table 6. Association between genetic susceptibility of white blood cell and PMD, stratified by depression and anxiety.^a^

|  | Women, N | PRS^b^ | RR (95% CI)^c^ | *P* |
| --- | --- | --- | --- | --- |
| Anxiety symptoms | | | | |
| With Anxiety | 65 | 0.31 ± 2.38 | 1.09 (0.91, 1.30) | 0.338 |
| Without Anxiety | 359 | 0.22 ± 2.39 | 1.08 (0.98, 1.19) | 0.104 |
| *P* for interaction |  |  |  | 0.682 |
| Depression symptoms | | | | |
| With Depression | 16 | 0.12 ± 2.20 | 0.82 (0.51, 1.33) | 0.438 |
| Without Depression | 286 | 0.27 ± 2.33 | 1.10 (1.00, 1.20) | 0.048 |
| *P* for interaction |  |  |  | 0.292 |
|  | | | | |
| No PMD | 1306 | -0.04 ± 2.41 | Reference |  |

^a^ PMD: Premenstrual disorders

^b^ PRS: Polygenic risk scores (mean ± standard deviation)

^c^ RR: Risk ratio; CI: Confidence interval. RRs were adjusted for four population principal components and age.

## Supplementary Table 7. Association between genetic susceptibility of white blood cell and physical symptom severity in PMD, stratified by depression and anxiety.^a^

|  | Women, N | PRS^b^ | Beta (95% CI)^c^ | *P* |
| --- | --- | --- | --- | --- |
| Anxiety symptoms | | | | |
| With Anxiety | 65 | 0.31 ± 2.38 | 0.06 (-0.21, 0.32) | 0.671 |
| Without Anxiety | 359 | 0.22 ± 2.39 | 0.04 (-0.07, 0.15) | 0.488 |
| *P* for interaction |  |  |  | 0.905 |
| Depression symptoms | | | | |
| With Depression | 16 | 0.12 ± 2.20 | 0.06 (-0.29, 0.41) | 0.747 |
| Without Depression | 286 | 0.27 ± 2.33 | 0.05 (-0.07, 0.17) | 0.408 |
| *P* for interaction |  |  |  | 0.421 |
|  | | | | |
| No PMD | 1306 | -0.04 ± 2.41 | 0.03 (-0.02, 0.08) | 0.223 |

^a^ PMD: Premenstrual disorders

^b^ PRS: Polygenic risk scores (mean ± standard deviation)

^c^ Beta: Beta coefficient; CI: Confidence interval. Betas represent the change in standardized physical symptom score per one standard deviation increase in PRS, adjusted for four population principal components and age.
